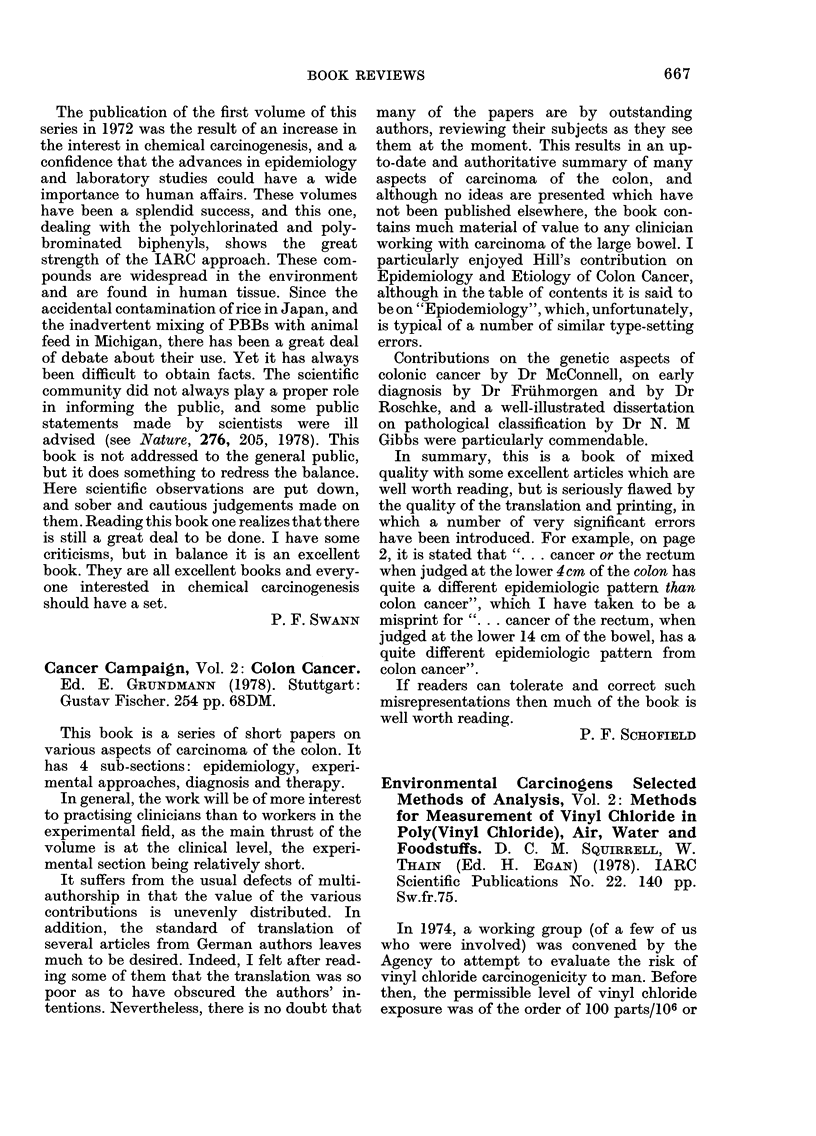# Cancer Campaign, Vol. 2: Colon Cancer

**Published:** 1979-10

**Authors:** P. F. Schofield


**Cancer Campaign,** Vol. 2: **Colon Cancer.** Ed. E. GRUNDMANN (1978). Stuttgart: Gustav Fischer. 254 pp. 68DM.

This book is a series of short papers on various aspects of carcinoma of the colon. It has 4 sub-sections: epidemiology, experimental approaches, diagnosis and therapy.

In general, the work will be of more interest to practising clinicians than to workers in the experimental field, as the main thrust of the volume is at the clinical level, the experimental section being relatively short.

It suffers from the usual defects of multi-authorship in that the value of the various contributions is unevenly distributed. In addition, the standard of translation of several articles from German authors leaves much to be desired. Indeed, I felt after reading some of them that the translation was so poor as to have obscured the authors' intentions. Nevertheless, there is no doubt that many of the papers are by outstanding authors, reviewing their subjects as they see them at the moment. This results in an up-to-date and authoritative summary of many aspects of carcinoma of the colon, and although no ideas are presented which have not been published elsewhere, the book contains much material of value to any clinician working with carcinoma of the large bowel. I particularly enjoyed Hill's contribution on Epidemiology and Etiology of Colon Cancer, although in the table of contents it is said to be on "Epiodemiology", which, unfortunately, is typical of a number of similar type-setting errors.

Contributions on the genetic aspects of colonic cancer by Dr McConnell, on early diagnosis by Dr Frühmorgen and by Dr Roschke, and a well-illustrated dissertation on pathological classification by Dr N. M Gibbs were particularly commendable.

In summary, this is a book of mixed quality with some excellent articles which are well worth reading, but is seriously flawed by the quality of the translation and printing, in which a number of very significant errors have been introduced. For example, on page 2, it is stated that ". . . cancer *or* the rectum when judged at the lower *4cm* of the *colon* has quite a different epidemiologic pattern *than* colon cancer", which I have taken to be a misprint for ". . . cancer of the rectum, when judged at the lower 14 cm of the bowel, has a quite different epidemiologic pattern from colon cancer".

If readers can tolerate and correct such misrepresentations then much of the book is well worth reading.

P. F. SCHOFIELD